# Microbial ecology of the newly discovered serpentinite-hosted Old City hydrothermal field (southwest Indian ridge)

**DOI:** 10.1038/s41396-020-00816-7

**Published:** 2020-11-02

**Authors:** Aurélien Lecoeuvre, Bénédicte Ménez, Mathilde Cannat, Valérie Chavagnac, Emmanuelle Gérard

**Affiliations:** 1 Université de Paris, Institut de physique du globe de Paris, CNRS UMR 7154, Paris, France; 2 Université de Toulouse, Géosciences Environnement Toulouse, CNRS UMR 5563, Toulouse, France

**Keywords:** Microbial ecology, Metagenomics, Biofilms

## Abstract

Lost City (mid-Atlantic ridge) is a unique oceanic hydrothermal field where carbonate-brucite chimneys are colonized by a single phylotype of archaeal Methanosarcinales, as well as sulfur- and methane-metabolizing bacteria. So far, only one submarine analog of Lost City has been characterized, the Prony Bay hydrothermal field (New Caledonia), which nonetheless shows more microbiological similarities with ecosystems associated with continental ophiolites. This study presents the microbial ecology of the ‘Lost City’-type Old City hydrothermal field, recently discovered along the southwest Indian ridge. Five carbonate-brucite chimneys were sampled and subjected to mineralogical and geochemical analyses, microimaging, as well as 16S rRNA-encoding gene and metagenomic sequencing. Dominant taxa and metabolisms vary between chimneys, in conjunction with the predicted redox state, while potential formate- and CO-metabolizing microorganisms as well as sulfur-metabolizing bacteria are always abundant. We hypothesize that the variable environmental conditions resulting from the slow and diffuse hydrothermal fluid discharge that currently characterizes Old City could lead to different microbial populations between chimneys that utilize CO and formate differently as carbon or electron sources. Old City discovery and this first description of its microbial ecology opens up attractive perspectives for understanding environmental factors shaping communities and metabolisms in oceanic serpentinite-hosted ecosystems.

## Introduction

Serpentinization is the hydration of mantle-derived rocks that produces fluids enriched in molecular hydrogen (H_2_). These reducing fluids can react with inorganic carbon to form methane, formate, and other low molecular-weight hydrocarbons, and organic acids [1]. They discharge through submarine hydrothermal vents or springs located in ophiolites where they provide energy and carbon sources for microbial life, while mixing with oxidized seawater or meteoric fluids [2, 3].

Oceanic serpentinite-hosted hydrothermal fields are found at (ultra)slow spreading ridges where mantle rocks are exhumed by tectonic processes and infiltrated by seawater [4, 5]. The emblematic Lost City hydrothermal field (LCHF, 30° N, mid-Atlantic ridge) is characterized by moderate temperature (up to 90–116 °C), metal- and CO_2_-depleted, high pH (9–11) fluids that actively discharge through carbonate-brucite chimneys [6, 7]. ‘Lost City’-type alkaline hydrothermal fields have also been discovered in the Mariana forearc [8] and at Prony Bay, New Caledonia [9]. These sites relate respectively to a subduction zone and an ophiolite. Fluid recharge at the Prony Bay hydrothermal field (PBHF) is mainly influenced by meteoric water, while the Shinkai Seep site of Mariana forearc and LCHF are fed by seawater.

Microbial communities sustained by serpentinization have recently been explored using next-generation sequencing and ‘-omic’ technologies, although oceanic hydrothermal systems remain poorly explored compared to terrestrial springs due to difficulties in discovering and sampling sites at great water depth. Microbial taxa inhabiting LCHF chimneys differ from other serpentinite-hosted ecosystems [10–12], and even from PBHF [13–16]. Nevertheless, LCHF shares some microbiological commonalities with these other serpentinization-influenced habitats, including its extremely low taxonomic diversity [10, 12]. LCHF biofilms are dominated by methane-, H_2_- and sulfur-metabolizing microorganisms with a unique archaeal phylotype of Methanosarcinales (the Lost City Methanosarcinales, LCMS) representing up to 81% of the community [11, 12, 17]. The LCMS metabolism can be versatile, and single species are capable of both hydrogenotrophic methanogenesis and anaerobic methanotrophy [18]. Sulfur-oxidizing Proteobacteria are represented by aerobic Rhodobacterales and Thiomicrospirales (formerly included in Thiotrichales). The LCHF microbial community is spatially heterogeneous, the LCMS being located inside the anoxic chimneys, while Thiomicrospirales colonize oxic/anoxic interfaces [12]. Methane-oxidizing bacteria requiring seawater O_2_ have been proposed as colonizers of the external parts of less active chimneys [12].

Despite differences in the composition of microbial communities retrieved at PBHF compared to LCHF, PBHF chimneys interestingly harbor Archaea related to LCMS and to another Methanosarcinales phylotype (The Cedars Methanosarcinales, TCMS) [15], identified in springs of The Cedars ophiolite (California, USA [19]). The dominant Bacteria in PBHF relate to anaerobic Firmicutes and aerobic or facultative anaerobic Chloroflexi, Proteobacteria, and Bipolaricaulota (formerly Acetothermia) [13–16]. The co-occurrence of aerobic and anaerobic metabolisms at both LCHF and PBHF has been attributed to variable influences of the hydrothermal discharge creating anoxic environments within chimneys and of oxidized seawater intrusions from outside the chimneys [12, 15]. However, since PBHF represents the only submarine analog of LCHF identified to date, the factors driving microbial ecology in these systems remain unclear.

Here, we describe the microbial ecology of the Old City hydrothermal field (OCHF), an oceanic ‘Lost City’-type hydrothermal site recently discovered along the southwest Indian ridge (SWIR) [20, 21]. Our study of the microbial diversity and its metabolic potential reveals significant taxonomic and metabolic heterogeneities between chimneys. By integrating mineralogical, geochemical, microimaging, microbial diversity, and metagenomic approaches, we hypothesize that the slow and diffuse hydrothermal discharge at Old City shapes the differences in microbial community composition and potential metabolisms between chimneys.

## Materials and methods

### Study site and sample processing

**Fig. 1:**
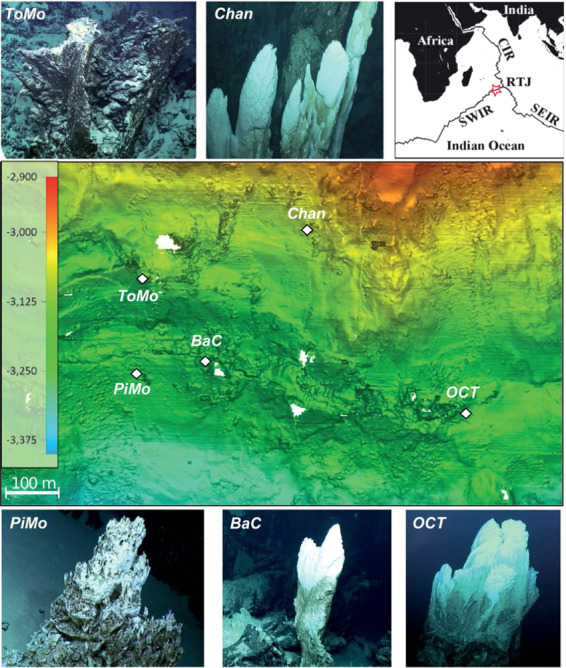
Microbathymetric map (in meters below sea level) of the Old City hydrothermal field (27° 50′ 6″ S to 64° 35′ 6″ E; southwest Indian ridge) and locations of vents (with dive photographs) sampled for this study using the remotely operated vehicle *Victor 6000*. Samples were collected from the recently formed white mineral deposit of potentially active chimneys (*BaC, Chan,* and *OCT*) or from apparently less active chimneys with dark coating made of biological mats and manganese deposits (*Tomo* and *Pimo)*. Details on samples can be found in Supplementary Table S1 and Supplementary methods. Videos of chimney sampling are also available at https://campagnes.flotteoceanographique.fr/campagnes/16002000/fr/. The red star in the context map reproduced from [22] indicates the region of the SWIR investigated during the ROVSMOOTH cruise. CIR central Indian ridge, RTJ Rodrigues triple junction, SEIR southeast Indian ridge.

OCHF was discovered at 27° 50′ 6″ S to 64° 35′ 6″ E during the ROVSMOOTH cruise (December 2016 to January 2017, R/V *Pourquoi pas?*, P.I. M. Cannat, IPGP [20, 21]). It is a serpentinite-hosted hydrothermal site located in a magma-poor area (less than 3% gabbros) of the eastern SWIR (Fig. 1) [5, 22]. This site is therefore particularly relevant to study the influence of serpentinization of mantle-derived rocks alone on microbial communities. OCHF sits at ~3,100 m below sea level (Supplementary Table S1) and consists of whitish vents (Fig. 1).

Five distinct chimney samples were collected (Fig. 1 and Supplementary methods). Three (i.e., *BaC, Chan,* and *OCT*) show recently formed deposits recognizable by their white color and highly porous and friable structure [10, 16]. In contrast, *PiMo* and *ToMo* have dark coating made of biological mats and manganese deposits. Videos of chimney sampling can be found at https://campagnes.flotteoceanographique.fr/campagnes/16002000/fr/ (Sampling videos for *BaC*, *Chan*, *OCT, PiMo* and *ToMo* are identified as 22773, 23014, 22788, 22772 and 23030, respectively).

Chimney samples were characterized for their mineralogical and elemental composition using X-ray diffraction (XRD) and inductively coupled plasma-mass spectrometry (ICP-MS), respectively. Microimaging was performed using confocal laser scanning microscopy (CLSM) and scanning electron microscopy (SEM). DNA was extracted from chimney samples following the phenol–chloroform protocol previously used for PBHF carbonate chimneys [14]. High-quality DNA extracts (up to 150 ng) were used for tag sequencing of the hypervariable region V4–V5 of the 16S rRNA-encoding genes and for shotgun metagenomics. All raw reads were submitted to the National Center for Biotechnology Information (NCBI) Sequence Read Archive under Bioproject accession number PRJNA556392. For all chimneys sampled (except *ToMo)*, fluids were also sampled as close as possible to putative chimney fluid outlets and in ambient seawater (Supplementary methods). Fluids were analyzed using inductively coupled plasma-optical emission spectrometry (ICP-OES). All analytical protocols and conditions are detailed in Supplementary methods, including results of control experiments (e.g., Supplementary Fig. S1).

### Amplicon filtering and microbial diversity analysis

High-quality sequences from tag sequencing were clustered into amplicon sequence variants (ASVs) by applying the minimum entropy decomposition algorithm [23] with default parameters but the minimum abundance set to 25 to remove noising features, while conserving relative diversities in each sample. Chimeras were identified in the representative ASV sequences with the *FindChimeras* algorithm implemented in the DECIPHER package for R [24, 25] and were then removed. Representative node sequences were submitted to the SILVAngs pipeline (https://www.arb-silva.de/ngs/) for taxonomic assignment of ASVs against the SILVA database release 132 [26]. As no DNA was amplified from procedural controls, we removed commonly identified contaminants [27] based on ASV taxonomic classification at the genus level (Supplementary Table S2). Taxa relative abundance in each sample was visualized using the ggplot2 package [28].

Filtered ASVs were imported in R version 3.6.0 and normalized using scaling factors calculated by the metagenomeSeq package [29]. Similarities and dissimilarities between communities were respectively evaluated using Bray–Curtis index with the anosim function and PERMANOVA implemented in the adonis function of the Vegan package [30], with 999 repetitions each time. Potential correlations between chimney microbial communities and associated mineralogical and elemental compositions were evaluated with Mantel tests based on Pearson’s product-moment or Spearman’s rank correlations implemented in the Vegan package [30].

### Metagenome analysis

Quality-filtered reads were assembled *de novo* with MEGAHIT v1.13 [31] with a minimum contig length of 1,000 bp and default parameters. For each sample, reads were mapped to the assembled contigs with Bowtie2 [32]. Mapped reads were counted for each gene with the *featureCounts* function of the Rsubread package for R [33]. Gene abundances were then normalized using the TMM normalization method implemented in the R package edgeR [34].

To infer the taxonomy of all genes predicted from metagenomes, their sequence similarities were evaluated against the non-redundant NCBI database using Diamond blastp tool [35] with an *e* value cutoff of 10^−5^. The most confident taxonomic rank assigned to each aligned gene sequence was predicted with the lowest common ancestor (LCA) algorithm implemented in MEGAN6 [36]. In addition to taxonomic annotation of all predicted genes, near full-length 16S rRNA-encoding genes were reconstructed for phylogenetic analysis (Supplementary methods). The relative abundance of bacterial and archaeal lineages in metagenomes was evaluated based on the relative abundance of (1) all retrieved genes, (2) 12 or 40 single-copy genes shared between all lineages as suggested by [37, 38], and (3) reconstructed near full-length 16S rRNA-encoding genes.

A set of marker genes (Supplementary Table S3) with normalized abundances was retrieved and imported in R for comparison and visualization using ggplot2 package [28]. Functional annotation of contigs was performed with the PROKKA pipeline [39] against the integrated custom database. In addition, protein sequences of genes identified by Prodigal [40] were submitted to GhostKOALA server (http://www.kegg.jp/ghostkoala/) to retrieve KEGG orthology [41] for each gene. Since some genes coding for hydrogenases are absent from the KEGG database, we also searched for hydrogenases domains [42, 43] in our metagenomes (Supplementary methods). All protein sequences of specific microbial lineages were finally retrieved and submitted to the GhostKOALA server in order to assess the completion of encoded metabolic pathways and to provide a more complete picture of the potential physiology of these lineages.

### Average carbon oxidation state of proteins predicted from metagenomes

The average carbon oxidation state (*Z*_C_) of protein sequences predicted from PROKKA [39] was calculated as described in [44]. Protein sequences were imported into R version 3.6.0 using the *read.fasta* function of the CHNOSZ package [45]. Since we used complete protein sequences derived from co-assembled contigs rather than reads, the amino acid counts of each protein were normalized by the abundance of the corresponding encoding genes obtained with Bowtie2 [32]. This normalization yields abundance-weighted values for each protein and allows comparison between samples. To account for intra-sample variations, sequences were randomly subsampled 100 times to generate a set of 200,000 amino acids on average for each sample, and were normalized by their length. *Z*_C_ values were finally computed using the *ZC(protein.formula(input))* function of R package CHNOSZ [45]. Results from subsampled sequences were used to calculate mean *Z*_C_ value and associated standard deviation for each sample, all plotted using ggplot2 package [28]. To assess differences between samples in *Z*_C_ mean values and variances, we respectively performed Welch’s *t*-test and pairwise Wilcoxon rank sum test in R version 3.6.0. Note that the overall approach only highlights the *Z*_C_ profile of encoded protein sequences and ignores potential differences between gene, mRNA and protein abundances [e.g., 46, 47].

## Results

### Fluid compositions

The compositions of fluids collected as close as possible to putative chimney fluid outlets and that of ambient seawater differ slightly from standard seawater (Supplementary Table S4). They show higher pH values and Mg, Ca, Na, and Li concentrations. The pH values of fluids collected from chimneys range from 7.88 (*OCT*) to 8.18 (*PiMo*). This pH value is slightly higher than that of ambient seawater (average pH 7.83), but their elemental compositions are similar.

### Hydrothermal chimneys’ elemental and mineralogical compositions

**Fig. 2:**
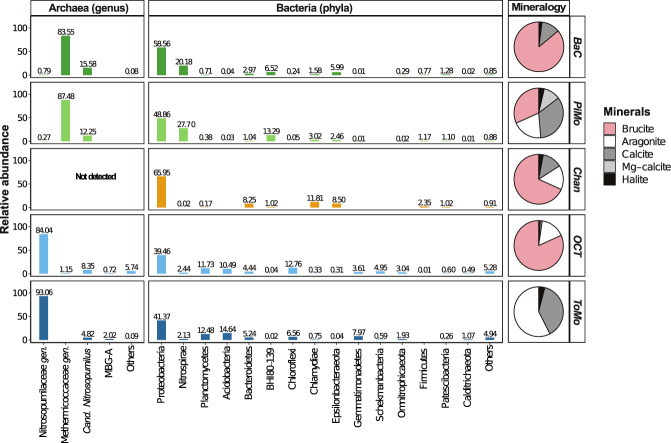
Relative abundance of taxa obtained from representative ASV sequences retrieved from Old City chimneys (at the genus and phylum level for Archaea and Bacteria, respectively and using the SILVA database release 132 [26]). Semi-quantitative X-ray diffraction results presenting the mineralogy of sampled chimneys are also shown as pie charts. The predominance of brucite is observed in most samples while *ToMo* is dominated by calcium carbonates (calcite and aragonite) and *PiMo* shows a similarly high relative content in calcite. *Cand. Nitrosopumilus* *Candidatus Nitrosopumilus*, MBG-A Marine Benthic Group-A, BHI80–139 candidate phylum BHI80–139/NPL-UPA2. Nitrosopumilaceae and Methermicoccaceae are two archaeal families with unclassified genera (*gen*.). “Others” includes all taxa that do not show a relative abundance higher than 1% in at least one chimney sample. It includes Actinobacteria and Bipolaricaulota (formerly Acetothermia) phyla.

OCHF chimney samples show variable chemical and mineralogical compositions. All have low metal concentrations (Supplementary Table S5), while the predominance of Mg and Ca depends on the chimney sampled. *PiMo* is the most enriched in Si and metals (i.e., Fe, Mn, Zn, and Ni). Semi-quantitative XRD results (Fig. 2) show that samples are mainly made of brucite and minor calcium carbonates, with the exception of *ToMo*, which is composed only of calcium carbonates (i.e., calcite and aragonite) and, to a lesser extent, *PiMo*, which shows a high relative content in calcite. *ToMo* sample is also enriched in Sr, in agreement with its high relative content in aragonite.

### Microscopic evidence for chimney microbial colonization

**Fig. 3:**
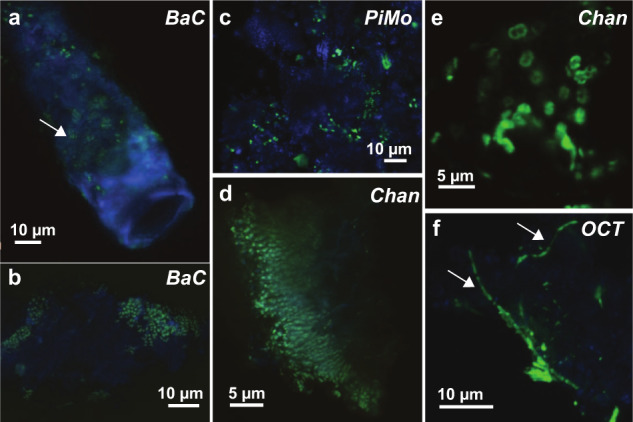
Confocal laser scanning microscopy images showing different microbial morphotypes in chimney samples. Rod-shaped cells (white arrows in **a**, **f**) and coccoidal cells (**a**–**d**) sometimes organized in filaments (**d**, **f**) are dominant in vent conduits where they attach to minerals here autofluorescing in blue (**a**–**c**). Diplococci, characteristic of methylotrophs, can be observed in *Chan* sample (**e**).

CLSM shows that chimneys are colonized by microorganisms grouped in clusters of cells attached to minerals (Fig. 3). Cell sizes range from 0.2 to 5 µm, with the smallest rod-shaped cells found in *BaC* and *PiMo* (0.5 × 0.2 µm; Fig. 3a–c), while the longest are found in *OCT* (5 × 1 µm; Fig. 3f). Dense biofilms consisting of small coccoidal cells (0.5 µm), sometimes organized in diplococci or tetrads, are present in *BaC* and *PiMo* (Fig. 3b, c). *Chan* is particularly enriched in diplococci (1–3 µm long; Fig. 3e) often organized in chains (Fig. 3d). Filamentous shapes are particularly abundant in *OCT* (30–35 µm long; Fig. 3f), but have also been observed in *BaC* (10–110 µm long).

Evidence of microbial colonization is less clear with SEM, possibly due to the widespread encrustation of cells in minerals (Supplementary Fig. S2). However, networks of thin carbonaceous filaments are ubiquitous (Supplementary Fig. S2a, e). By analogy to what has been observed in PBHF chimneys [16], they likely represent extracellular polymeric substances. Some OCHF chimneys also show the presence of bacteriomorphs (Supplementary Fig. S2b) or long carbon-rich filamentous structures (several tens of micrometers long) coated with nano- to microcrystalline brucite (Supplementary Fig. S2d), which could lead to the large mineralized filaments frequently observed (Supplementary Fig. S2c), as also reported in PBHF chimneys [16].

### DNA quantification and sequence filtering

DNA extractions led to concentration ranging from 0.06 to 4.20 µg/g of DNA per chimney samples. *OCT* and *ToMo* harbor relatively low DNA concentrations (0.06 and 0.21 µg/g, respectively) compared to other chimney samples. However, all produced high-quality amplicon sequences with an average of 148,217 and 82,906 quality filtered merged paired-end reads for Bacteria and Archaea, respectively (Supplementary Table S6). In addition, a total of 190,288,385 high-quality metagenomic paired-end reads were sequenced (Supplementary Table S6) and co-assembled to 391,941 contigs with an average length of 2,000 nucleotides.

### Microbial community composition from tag sequencing

Statistical analyses of similarities and dissimilarities show that the microbial communities of *BaC* and *PiMo* on the one hand and *OCT* and *ToMo* on the other hand are significantly similar while *Chan* presents a distinct community (PERMANOVA tests, *r*² = 0.67 and *p* < 0.001, respectively). However, Mantel tests suggest that microbial communities’ diversity is not correlated with bulk chimney mineralogy and chemistry (Supplementary Table S7).

The relative abundance of taxonomic groups identified from 16S rRNA-encoding gene amplicons (i.e., from ASV sequences) confirms heterogeneities between samples for both Bacteria and Archaea (Fig. 2). while Proteobacteria dominate all samples, the other dominant phyla differ strongly between chimney samples. Uncultured Thermodesulfovibrionia (Nitrospirae) and candidate phylum BHI80–139 (also known as NPL-UPA2) are abundant in *BaC* and *PiMo*. In contrast, Bacteroidetes, Chlamydiae, and Epsilonbacteraeota are relatively abundant in *Chan*, while Planctomycetes, Acidobacteria and Chloroflexi are only well represented in *OCT* and *ToMo*. The relative abundances of proteobacterial lineages also depend on samples (Supplementary Fig. S3). For Gammaproteobacteria, the Thiomicrospirales order prevails in *BaC* and *PiMo*, while the Steroidobacterales order is more abundant in *OCT* and *ToMo*. In contrast, Methylococcales-related sequences are 30 times more abundant in *Chan* compared to other chimney samples and at least three times more abundant than any other taxa in this sample. Alphaproteobacteria in *BaC*, *PiMo,* and *Chan* mostly group in the versatile Rhodobacterales order, while most of the alphaproteobacterial ASVs in *OCT* and *ToMo* belong to the Rhodovibrionales and Kordiimonadales orders (Supplementary Fig. S3). In *BaC*, *PiMo,* and *Chan*, deltaproteobacterial ASVs are mainly represented by the sulfate reducing Desulfobacterales order, while Deltaproteobacteria are less abundant in *OCT* and *ToMo*.

Archaea are not detected in *Chan* using tag sequencing. As observed for Bacteria, *BaC*, and *PiMo* Archaea are highly similar with the dominance of ASVs related to the Methermicoccaceae family from the Methanosarcinales order (Fig. 2). In contrast, the most abundant taxon in *OCT* and *ToMo* corresponds to unclassified Nitrosopumilaceae. Only *Candidatus Nitrosopumilus* is shared between samples at comparable abundance.

### Relative proportion of Archaea and Bacteria in metagenomes

Lineage relative abundance profiles have also been established from metagenomes following four different approaches (Supplementary Fig. S4). They all show that Archaea are less abundant in *BaC* and *PiMo* with Euryarchaeota, and in particular Methanosarcinales, representing only 2–4% of all genes considered. In contrast, Thaumarchaeota, including the Nitrosopumilaceae family, are highly abundant in *OCT* and *ToMo*, representing 17–40% and 11–28% of all genes, respectively. The mostly unclassified Euryarchaeota observed in *Chan* and *ToMo* are very low in abundance (<1% of all genes).

### Phylogenetic analysis

**Fig. 4:**
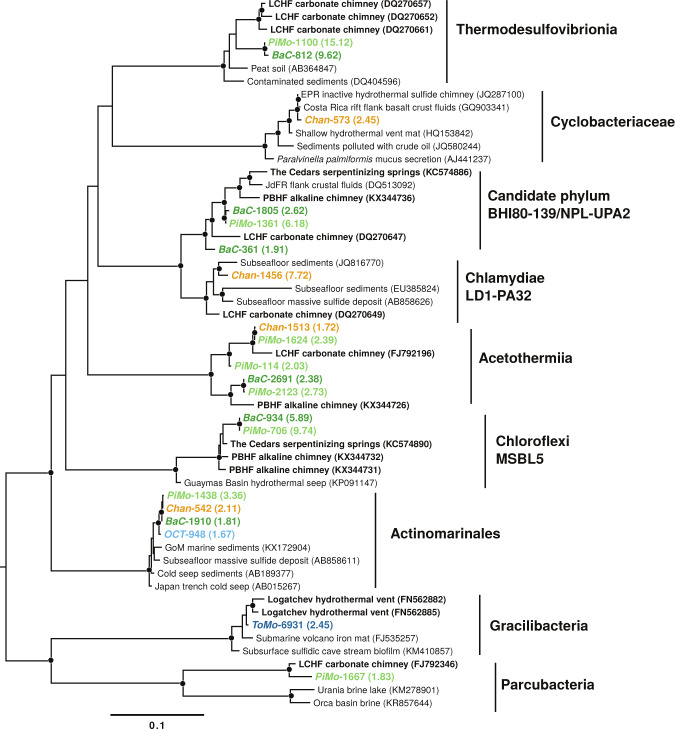
Maximum likelihood phylogenetic tree of Nitrospirae (Thermodesulfovibrionia), Bacteroidetes (Cyclobacteriaceae), candidate phylum BHI80–139/NPL-UPA2, Chlamydiae, Acetothermia (Acetothermiia), Chloroflexi, Actinobacteria (Actinomarinales) and Patescibacteria (Gracilibacteria and Parcubacteria) based on near full-length 16S rRNA-encoding gene reconstructed from metagenomic reads. To highlight phylotype diversity, only abundant sequences (>1.5%) are represented. Sequences from this study are colored with respect to chimney samples (Supplementary Table S1) with normalized relative abundance in brackets (%). Environmental clone accession numbers are indicated in brackets with serpentinization-related ecosystems in bold. They correspond to the closest hits to the near full-length 16S rRNA-encoding gene sequences reported in this study using the SILVA database release 132 [26]. Bootstrap values over 70% support based on 1,000 replicates are indicated by black dots on their respective nodes. LCHF Lost City hydrothermal field, PBHF Prony Bay hydrothermal field, EPR East Pacific rise, JdFR Juan de Fuca ridge, GoM, Gulf of Mexico.

Based on the metagenomic datasets, we have reconstructed 364 high-quality near full-length 16S rRNA-encoding gene sequences. As shown in Fig. 4 and Supplementary Figs. S5–S7, these sequences group with all the dominant taxonomic groups highlighted by tag-sequencing community analysis (Fig. 2) despite differences in abundance (e.g., Actinobacteria and Acetothermia which are much more abundant in metagenomes; Supplementary Fig. S4).

*BaC* and *PiMo* share many similarities regarding their Bacteria and Archaea phylogenetic affiliations. Many Bacteria of these chimney samples closely group with clones retrieved from LCHF (Fig. 4 and Supplementary Figs. S5 and S6) including Thermodesulfovibrionia (Nitrospirae), dominant *Thiomicrorhabdus* (Gammaproteobacteria), and some *Sulfurovum* (Epsilonbacteraeota) and *Desulfobulbus* (Deltaproteobacteria). Sequences grouping with Chloroflexi Dehalococcoidia (MSBL5) relate to environmental clones from PBHF and The Cedars. Interestingly, three reconstructed near full-length 16S rRNA-encoding genes group with candidate phylum BHI80–139/NPL-UPA2 in a cluster formed by clones from LCHF, PBHF, The Cedars and Baby Bare seamount on the Juan de Fuca ridge flank (Fig. 4). Two near full-length 16S rRNA-encoding gene sequences from *BaC* and *PiMo* closely relate to TCMS (Supplementary Fig. S7).

Numerous bacterial and archaeal near full-length 16S rRNA-encoding genes from *OCT* and *ToMo* closely group with environmental clones from deep marine sediments, polymetallic nodules, and sometimes from the ultramafic rock-hosted Logatchev hydrothermal field. In contrast, *Chan* includes bacterial phylotypes grouping with more diverse environments, including macrofauna bionts, in addition to marine sediments and hydrothermal vents (Fig. 4 and Supplementary Figs. S5–S7).

### Metabolic potential for C1 compounds

**Fig. 5:**
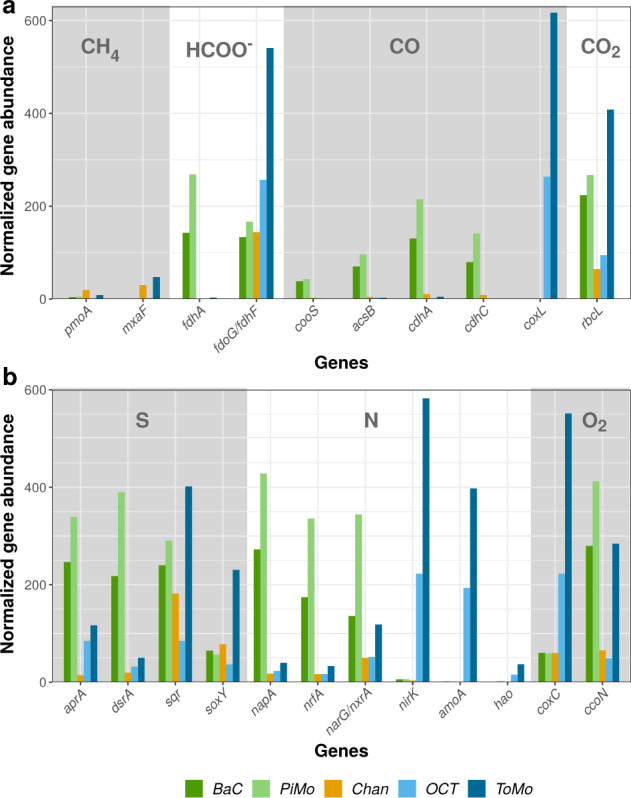
Normalized abundance of key genes in chimney metagenomes according to PROKKA [39] and KEGG orthology [41] annotations. **a** Genes involved in C1 compounds’ metabolisms. **b** Genes involved in sulfur, nitrogen, and oxygen metabolisms. Each gene and its function is described in Supplementary Table S3.

**Fig. 6:**
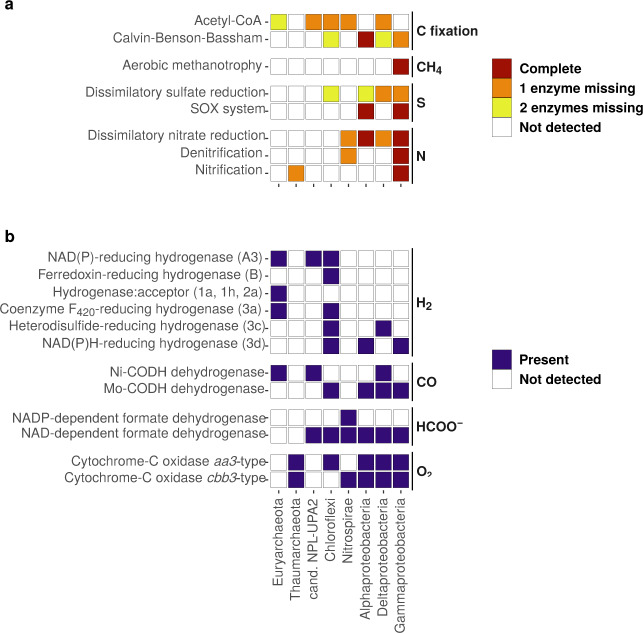
Overview of metabolic pathways’ completion and of the presence of key enzyme-encoding genes in dominant microbial lineages of chimney metagenomes. Colored boxes highlight the presence and completion of metabolic pathways (**a**) or key genes coding for the enzyme indicated (**b**) as determined from KEGG module and orthology [41]. Metabolic pathway that are missing half of all required genes are considered undetected. Nitrogen fixation and methanogenesis are not indicated due to their absence or poor completion in all microbial lineages investigated.

Most of the key metabolic genes involved in C1 compounds’ metabolisms (Supplementary Table S3) are detected in all five metagenomes, but their abundance varies similarly to taxonomic diversity (Fig. 5a). However, the key gene coding for the methyl-coenzyme M reductase (*mcrA*) involved in methanogenesis is not detected in any metagenome, despite the presence of Methanosarcinales in *BaC* and *PiMo*. No complete or nearly complete methanogenesis pathways (including those involving CO_2_, acetate, methanol or methylamine) can be detected in the OCHF archaeal lineages. In addition, the genes involved in aerobic methanotrophy (*pmoA* and *mxaF*) are detected only at low abundances compared to other genes, with *Chan* and *ToMo* showing the highest abundance (Fig. 5a). A complete metabolic pathway for aerobic methanotrophy can only be identified for Gammaproteobacteria (Fig. 6a).

Interestingly, genes related to formate and CO metabolisms show strong differences between chimney samples. The genes encoding NADP-dependent formate dehydrogenase (*fdhA*), anaerobic CO dehydrogenase (Ni-CODH; *cooS* and *cdhA*) and acetyl-coenzyme A (CoA) synthase (ACS; *acsB* and *cdhC*) are particularly enriched in *BaC* and *PiMo* (Fig. 5a). These genes can be involved in carbon fixation through the reductive acetyl-CoA pathway. This pathway is nearly complete for candidate phylum BHI80–139/NPL-UPA2, Chloroflexi, Nitrospirae, and Deltaproteobacteria (Fig. 6a), these lineages being abundant in *BaC* and *PiMo* (Supplementary Fig. S4). In contrast, the NAD-dependent formate dehydrogenase-encoding gene (*fdoG/fdhF*) is abundant in all samples, with *OCT* and *ToMo* showing the highest abundance (Fig. 5a). This gene is detected in all dominant bacterial lineages (Fig. 6b). *OCT* and *ToMo* also show a high abundance of *coxL* gene encoding a CO dehydrogenase (Mo-CODH) involved in aerobic carboxydotrophy (Fig. 5a). The Mo-CODH-encoding gene is identified in proteobacterial lineages as well as in Chloroflexi (Fig. 6b), which are relatively well represented in *OCT* and *ToMo* (Supplementary Fig. S4).

The *rbcL* gene coding for the ribulose-bisphosphate carboxylase, involved in the aerobic Calvin–Benson–Bassham cycle, is detected in all samples but is dominant in *BaC*, *PiMo*, and *ToMo* (Fig. 5a). The Calvin–Benson–Bassham cycle is complete in Alphaproteobacteria and nearly complete in Gammaproteobacteria (Fig. 6a).

### Potential sulfur, nitrogen, and oxygen metabolisms

Although less abundant in *OCT*, the sulfide:quinone oxidoreductase-encoding gene (*sqr*) shows high abundance in all metagenomes, suggesting, along with the ubiquitous presence of the *soxY* gene, that oxidation of reduced-sulfur species could be an important metabolism in all chimneys (Fig. 5b). The SOX system, involved in thiosulfate oxidation, is detected only in Alpha- and Gammaproteobacteria (Fig. 6a). In contrast, genes potentially involved in dissimilatory sulfate and sulfite reduction (*aprA* and *dsrA*) and nitrate and nitrite reduction to ammonia NH_3_ (*napA* and *nrfA*) or cytoplasmic nitrate reduction (*narG*) are enriched in *BaC* and *PiMo* (Fig. 5b). The dissimilatory sulfate reduction pathway is nearly complete for Delta- and Gammaproteobacteria, while the dissimilatory nitrate reduction pathway is complete or nearly complete for all proteobacterial lineages and Nitrospirae (Fig. 6a). The ammonia monooxygenase-encoding gene (*amoA*) and, to a lesser extent, the hydroxylamine dehydrogenase-encoding gene (*hao*), involved in nitrification (i.e., ammonia oxidation), are particularly enriched in *OCT* and *ToMo* (Fig. 5b). Potential for nitrification is found in Gammaproteobacteria and Thaumarchaeota lineages (Fig. 6a) while the *hao* gene is not detected in OCHF Thaumarchaeota. The *nirK* gene is also abundant in *OCT* and *ToMo* metagenomes (Fig. 5b). Interestingly, *nirK* has been identified in Thaumarchaeota, suggesting a potential role of this lineage in nitrogen monoxide-dependent hydroxylamine oxidation [48], in addition to denitrification for other lineages (Fig. 6a).

Genes involved in aerobic respiration are also represented in OCHF metagenomes with predominantly the *ccoN* gene coding for cytochrome-C oxidase *cbb3-*type in *BaC* and *PiMo*, while *OCT* and *ToMo* are dominated by the cytochrome-C oxidase *aa3*-type-encoding gene (*coxC*) (Fig. 5b). Both genes are present in all proteobacterial lineages and in Thaumarchaeota (Fig. 6).

### Potential hydrogen metabolisms

**Fig. 7:**
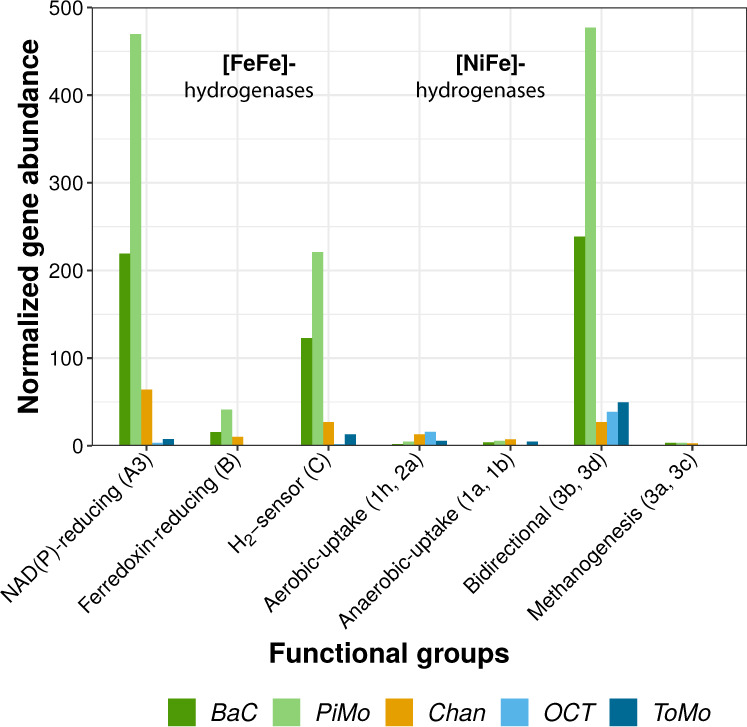
Normalized abundance of genes encoding hydrogenases. The different groups or subgroups [42, 43] are identified in brackets. Each group of hydrogenases identified in our metagenomic data and its functions is described in Supplementary Table S3.

Genes encoding oxygen sensitive [FeFe]-hydrogenases groups A, B, and C are one to two orders of magnitude more abundant in *BaC* and *PiMo*, but also more abundant in *Chan* compared to *OCT* and *ToMo* (Fig. 7). The enzyme and homology annotations of [FeFe]-hydrogenases group A reveal similarities with the *hndD* gene coding for NADP^+^-reducing hydrogenases, which may belong to subgroup A3 [42, 43]. This gene is detected in Euryarchaeota, in the candidate phylum BHI80–139/NPL-UPA2 and in Chloroflexi (Fig. 6b). In contrast, the gene encoding [FeFe]-hydrogenases group B shows similarities with the *hydA* gene and is only observed in Chloroflexi (Fig. 6b).

Apart from the oxygen tolerant subgroups 3b and 3d involved in the reversible oxidation of H_2_, genes encoding [NiFe]-hydrogenases, including those involved in anaerobic uptake of H_2_, are one order of magnitude less abundant in OCHF metagenomes compared to [FeFe]-hydrogenase-encoding genes (Fig. 7). The *hox* gene encoding [NiFe]-hydrogenases subgroup 3d is found in Chloroflexi, and Alpha- and Gammaproteobacteria (Fig. 6b). Genes coding for [NiFe]-hydrogenases subgroups 3a and 3b, involved in hydrogenotrophic methanogenesis, are scarce in all metagenomes and relate to Chloroflexi and Deltaproteobacteria (Figs. 6b and 7).

### Carbon oxidation state of predicted proteomes

**Fig. 8:**
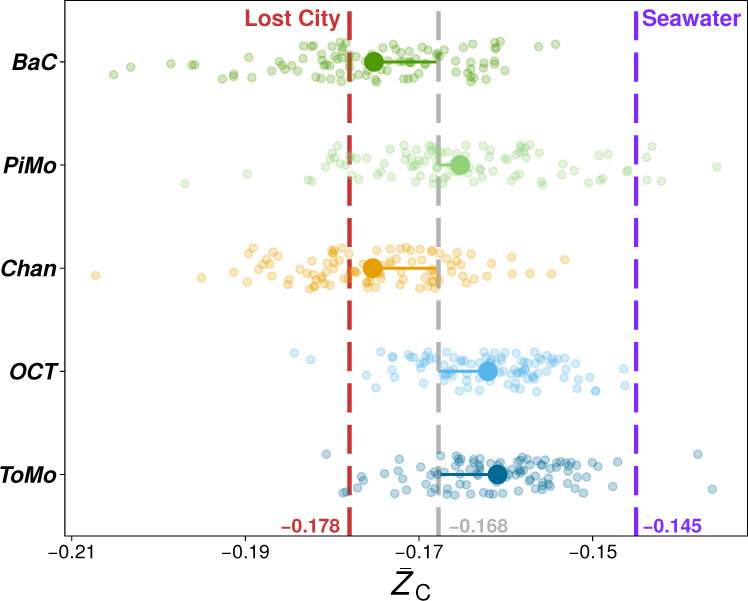
Distribution of average carbon oxidation state (*Z*_*C*_) estimated for the subsampled proteins predicted from metagenomes. For each chimney sample, the vertical distribution was artificially computed to avoid overlap. The gray dashed vertical line highlights mean *Z*_C_ value (−0.168) obtained at OCHF for all metagenomes and dark dots indicate the mean *Z*_C_ value for each predicted proteome with distance from the mean *Z*_C_ value. *Z*_C_ values calculated by [44] for Lost City hydrothermal chimneys (red dashed line) and ambient seawater near the Axial seamount, north east Pacific Ocean (blue dashed line), are indicated for comparison. Cand. candidate phylum.

All OCHF predicted proteomes are, on average, relatively reduced compared to *Z*_C_ values calculated for predicted proteomes from ambient seawater near the Axial seamount, north east Pacific Ocean, but more oxidized than the proteomes predicted for LCHF chimneys [44] (Fig. 8). Nevertheless, *Z*_*C*_ mean values and variances differ significantly from sample to sample. In contrast to their microbial diversity and metabolic potential, *Z*_*C*_ mean values and variances of *BaC* and *PiMo* (−0.1752 ± 0.010 and −0.1653 ± 0.011, respectively) are significantly different (*p* < 0.001). *Chan* (mean *Z*_C_ = −0.1753 ± 0.009) and *BaC* show highly similar (*p* = 0.914) reduced proteomes compared to other samples (*p* < 0.001). *OCT* and *ToMo* predicted proteomes are significantly more oxidized (mean *Z*_C_ = −0.1620 ± 0.007 and −0.1609 ± 0.008, respectively) and similar (*p* = 0.310), while being different from *PiMo* (*p* = 0.015 and 0.002 for *OCT* and *ToMo*, respectively).

## Discussion

### Environmental conditions

At OCHF, no focused hydrothermal discharges in the form of plumes or shimmering fluids have been observed, suggesting that the hydrothermal fluid flow is extremely slow and diffuse. This diffuse flow made it impossible with the equipment available during the ROVSMOOTH cruise to sample end-member hydrothermal fluid properly without seawater influence [49]. Nevertheless, fluids sampled close to putative chimney fluid outlets reveal slightly higher pH values than ambient seawater near vents associated with ultramafic rocks [50] and hydrothermal fluids of the serpentinization-related Von Damm, Rainbow, or Logatchev hydrothermal fields [3] (Supplementary Table S4). Although lower than pH values measured at LCHF, PBHF and other alkaline serpentinite-hosted ecosystems [3], these pH values, as well as the presence of brucite and carbonate chimneys, highlight the alkaline nature of Old City hydrothermal fluids. Their slight enrichment in Mg, Ca, Na, and Li compared to standard seawater (Supplementary Table S4) could be related to the dissolution of brucite and carbonate minerals during sampling or to the influence of alkaline hydrothermal fluids [9].

Like LCHF, PBHF and the Mariana forearc Shinkai Seep [6, 8, 9, 16], most of the OCHF chimney samples are mainly composed of brucite with minor aragonite and/or calcite (Fig. 2). Since brucite is only stable at pH above 10 [9], ref. [51] proposed that LCHF chimneys should initially consist of brucite and aragonite, gradually replaced by calcite as hydrothermal activity decreases and seawater influence increases. The increasing influence of seawater causes a decrease in fluid pH, hence dissolving brucite. The resulting increase in pH causes carbonate ions from seawater to precipitate with divalent cations such as Ca^2+^ and Mg^2+^ from hydrothermal fluids or seawater. This is consistent with the dark coating made of biological mats and manganese deposits reported for *ToMo* and *PiMo* chimneys (Fig. 1), suggesting that these may be less active compared to the whitish, porous, and friable structures associated with recent hydrothermal deposits that characterize active chimneys [10, 16]. Indeed, these samples contain higher relative proportions of calcium carbonates than the other chimney samples that are dominated by brucite. However, while brucite in absent from *ToMo*, its high relative content in *PiMo* suggests that this chimney is still active, as also supported by its microbial community composition.

Overall, the mineralogy of our samples, indicative of exposure to hydrothermal fluid versus seawater chemistry, suggests that recent mineral deposits where brucite dominates could have been formed from discharging alkaline hydrothermal fluids. In addition, the dominance of brucite could indicate a higher pH for the hydrothermal fluids compared to the values reported in Supplementary Table S4.

### Variability of local environmental conditions in chimneys

Recent studies have shown a strong correlation between the decrease in *Z*_C_ values of predicted proteomes and increased alkaline and reducing conditions [44, 52]. As observed in sediments and other hydrothermal fields [53, 54], the diffuse fluid flow at OCHF can lead to highly heterogeneous geochemical conditions with mixed but variable influences of reducing hydrothermal fluids and oxygenated seawater intrusions, and thus to variable *Z*_*C*_ values. This is consistent with the carbon oxidation state estimated for OCHF chimneys from predicted proteomes that shows significant variability within and between samples (Fig. 8). This highlights different environmental conditions at the chimney scale or across the entire hydrothermal field. Compared to *Z*_C_ values of predicted proteomes from ambient seawater near hydrothermal vents [44], the *Z*_*C*_ values estimated for OCHF are lower (Fig. 8), overall suggesting more reducing and alkaline conditions on average. These reducing and alkaline conditions are consistent with the predictions made above from the mineralogy, except for *OCT* which has *Z*_*C*_ values similar to those of *ToMo* despite a higher relative content in brucite. This suggests for *OCT* a greater influence of seawater intrusions than in other chimney samples. *OCT* was sampled at the top of the OCHF tallest chimney (i.e., 52 m above seafloor) and is located away from the other sampled chimneys. This raises questions about the influence of vent geographical distribution as well as subseafloor architecture and associated hydrodynamics on chimney microbial ecology and prevailing environmental conditions.

### Methane metabolisms

Potential methanogenic Archaea (i.e., Methanosarcinales) are less represented in the studied chimneys than Bacteria (Supplementary Fig. S4). Moreover, complete methanogenesis pathways are missing in OCHF Euryarchaeota. Although these results could be attributed to the limitations of LCA annotations, methanogens may not be dominant at OCHF. This recalls ophiolite ecosystems where potential methanogenic archaea are generally rare or absent, whereas they flourish within active LCHF chimneys [11, 55–57]. Nevertheless, our study covers only a small portion of chimneys and we cannot exclude the possibility that Methanosarcinales are present at higher abundance than what we observe at OCHF.

Interestingly, *Chan* presents the most reduced predicted proteomes (Fig. 8). This sample is dominated by potential aerobic methane- or methyl-oxidizing bacteria, such as *Methyloprofundus*, which may appear as diplococci in *Chan* samples (Fig. 3e) [58]. This is supported by the highest abundance in *Chan* metagenome of genes involved in aerobic methanotrophy (*pmoA* and *mxaF*), and by the fact that complete aerobic methane oxidation pathway is only found in Gammaproteobacteria, which also dominate *Chan* microbial community (Figs. 5 and 6 and Supplementary Fig. S4). Potential aerobic methanotrophs among Methylococcaceae have been reported in several oceanic and ophiolitic serpentinite-hosted ecosystems, including LCHF [11, 55–57]. Although this microbial lineage requires oxygen from seawater, it may also be sustained at OCHF by reduced carbon sources including methane potentially derived from abiotic organic synthesis associated with serpentinizing environments and leading to reduced proteomes, as observed for *Chan*.

### Carbon monoxide and formate metabolisms

Like methane, CO and formate can also derive from abiotic organic synthesis promoted in such environments [59, 60]. Since CO_2_ is usually depleted in low-temperature serpentinization-influenced hydrothermal fluids due to the associated alkaline pH [3], both abiotic CO and formate can represent valuable alternative electron donors and carbon sources. Genes involved in anaerobic or aerobic CO- and formate-based metabolisms are abundant in OCHF metagenomes but they show different profiles between samples (Fig. 5a) suggesting different utilization of CO and formate. While *BaC* and *PiMo* are enriched in genes coding for anaerobic Ni-CODH (*cooS* or *cdhA*) and NADP-dependent formate dehydrogenase (*fdhA*), *OCT* and *ToMo* metagenomes show high abundance of *coxL* gene coding for aerobic Mo-CODH, in agreement with the associated microbial ecology. As proposed for the Samail ophiolite [52], this may be related to the different environmental conditions (i.e., redox and pH) that prevail in OCHF chimneys (e.g., Fig. 8).

The NAD-dependent formate dehydrogenase-encoding gene (*fdoG/fdhF*) is abundant in all samples (Fig. 5a) and is widespread among OCHF bacterial lineages (Fig. 6) pointing to formate to be an important electron donor at OCHF. It should be noted that NAD-dependent formate dehydrogenase could serve as an alternative to the NADP-dependent formate dehydrogenase in putative acetogens [61]. Moreover, together with genes encoding acetyl-CoA synthase (*acsB* or *cdhC*), the Ni-CODH-encoding genes are known to be involved in acetogenesis through the carbonyl branch of the oxidative or reductive acetyl-CoA pathway [62]. The abundance of Ni-CODH-encoding genes in *BaC* and *PiMo* (Fig. 5a) suggests high metabolic potential for CO-utilizing acetogenesis that could be related to the candidate phylum BHI80–139/NPL-UPA2, Chloroflexi, Nitrospirae, and Deltaproteobacteria (Fig. 6), all of which being abundant in these chimneys. These taxa are associated with LCHF, PBHF, or The Cedars phylotypes (Fig. 4 and Supplementary Fig. S6), overall suggesting that acetogenic bacteria could be widespread in serpentinite-hosted ecosystems. Nevertheless, the Ni-CODH-encoding gene (*cooS* or *cdhA*) is not detected in OCHF Chloroflexi and Nitrospirae. Like for the lack of genes for methanogenesis, it could be explained by the poor specificity of LCA taxonomic annotation.

Conversely, *coxL* coding for a Mo-CODH involved in aerobic oxidation of CO is abundant in *OCT* and *ToMo* metagenomes (Fig. 5a). It has been reported for the Tablelands ophiolite (Canada) that CO carried by serpentinization-derived reducing fluids could serve as electron donor rather than for biomass synthesis when mixing with oxidized surface waters [63]. Accordingly, CO may represent a valuable electron donor in *OCT* and *ToMo* chimneys where *coxL* is abundantly detected in all proteobacterial lineages, with Alpha- and Gammaproteobacteria being abundant, and in Chloroflexi (Fig. 6). This is consistent with the low abundance of putative acetogens and the significantly higher *Ź*_*C*_ values estimated for *OCT* and *ToMo* compared to other chimneys (Fig. 8), overall suggesting that in *OCT* and *ToMo*, in addition to formate, CO could be oxidized by dominant Alpha- and/or Gammaproteobacteria rather than being used as carbon source for acetogenesis as in *BaC* and *PiMo*.

### Hydrogenases and potential microbial adaptation

As H_2_ solubility and mobility decreases with salinity and lower temperature [64], an increased influence of seawater intrusions, supported by the mineralogy of these chimneys, may lead to the depleted abundance in hydrogenases observed in *OCT* and *ToMo* (Fig. 7). Conversely, genes encoding [FeFe]-hydrogenases group A3 (*hndD*) and [NiFe]-hydrogenases groups 3b and 3d (*hoxH*) are particularly enriched in *BaC* and *PiMo* where a high metabolic potential for acetogenesis related to candidate phylum BHI80–139/NPL-UPA2, Chloroflexi, Nitrospirae, and Deltaproteobacteria has been suggested. [FeFe]-hydrogenase group A3 could be associated with acetogenesis by providing reduced NADH and ferredoxin from the oxidation of H_2_ [43, 65]. Genes encoding [FeFe]-hydrogenases group A3 are consistently found in some of the potential acetogens dominating in *BaC* and *PiMo* and in Euryarchaeota (Fig. 6). Although no hydrogenase-encoding genes has been reported in the genome of the candidate phylum BHI80–139/NPL-UPA2 retrieved at The Cedars [66], it should be noted that *hndD* presents homologies with the *nuoF* gene coding for a subunit of the respiratory complex I and identified in this genome [65]. Given that certain hydrogenases have been proposed as potential precursors of this complex [67] and that it is unlikely that the genome of the candidate phylum BHI80–139/NPL-UPA2 encodes a complete respiratory complex I, we suggest that the identified *nuoF* gene may instead encode a [FeFe]-hydrogenases group A3, hence supporting acetogenic pathway for candidate phylum BHI80–139/NPL-UPA2.

[NiFe]-hydrogenases groups 3b and 3d have been proposed to serve as an electron valve to regulate intracellular oxidation state [43]. Under anoxic reducing conditions, these hydrogenases can be involved in H_2_ fermentative evolution from NAD(P)H produced, for example, by formate oxidation. Under oxic conditions, they are associated with H_2_ uptake for energy production, in association with the respiratory complex I, or with biosynthesis, which requires NAD(P)H [67]. [NiFe]-hydrogenases groups 3b and 3d could therefore be versatile enough to cope with variable mixing of reducing hydrothermal fluids and oxygenated seawater. At OCHT, [NiFe]-hydrogenase groups 3b and 3d are detected in Chloroflexi phylotypes (Fig. 6), which group with Chloroflexi from PBHF and The Cedars (Fig. 4), two sites characterized by strongly reducing conditions where these phylotypes are also abundant [13, 14, 19]. Moreover, [NiFe]-hydrogenase-encoding genes were also detected in reconstructed genomes of LCHF Chloroflexi [68]. These [NiFe]-hydrogenases-encoding genes are also found in OCHF Alpha- and Gammaproteobacteria (Fig. 6). Formate dehydrogenase-encoding genes and nearly complete Calvin–Benson–Bassham cycle are also detected in these lineages, suggesting that OCHF Alpha- and Gammaproteobacteria may have the metabolic potential for fermentative formate oxidation as well as CO_2_ fixation with H_2_ oxidation [69].

### Other metabolisms

At LCHF, the exterior of less active or inactive chimneys is dominated by Proteobacteria that metabolize oxidized compounds derived from seawater (i.e., O_2,_ CO_2_, sulfate, and nitrate) [10, 11]. Similarly, at OCHF, proteobacterial lineages show metabolic capabilities for aerobic respiration, CO_2_ fixation through the Calvin–Benson–Bassham cycle, as well as sulfur and nitrogen metabolisms (Fig. 6). The abundance of Deltaproteobacteria and genes involved in dissimilatory sulfate reduction in *BaC* and *PiMo* (Fig. 5b and Supplementary Fig. S4) suggest that this metabolism dominates in these chimney samples. However, it should be noted that genes involved in dissimilatory sulfate reduction could mediate the reverse reaction, i.e., sulfur oxidation to sulfate likely to be operated by Alpha- and Gammaproteobacteria, considering that the SOX system is complete in these lineages (Fig. 6). In addition to sulfate contained in seawater [10, 11], sulfate may also be enriched in hydrothermal fluids due to extensive subseafloor circulation of oxidized seawater during peridotite exhumation, which could lead to local oxidation of sulfides [70]. Sulfur-oxidizing bacteria, such as aerobic proteobacterial Rhodobacterales, Thiomicrospirales, Thiotrichales (Fig. 2 and Supplementary Fig. S3) and related metabolic genes are widespread in all OCHF chimney samples. Most of these lineages are also common to LCHF (Supplementary Fig. S5 and S6). Reduced-sulfur compounds could be possibly derived from both biotic (i.e., from sulfate reducing bacteria) or abiotic components in the hydrothermal fluid.

Both Nitrospirae and Gammaproteobacteria could be involved in nitrate reduction through dissimilatory nitrate reduction or denitrification (Fig. 6). However, although such metabolisms have been reported to be possible in other serpentinite-hosted ecosystems [55–57], a low concentration of nitrate is usually documented in such systems. *OCT* and *ToMo* are dominated by Thaumarchaeota, which are more likely to originate from deep seawater where they are widespread [71], than from hydrothermal fluids. The relative enrichment in ammonia monooxygenase-encoding genes (*amoA*) in *OCT* and *ToMo* suggests that Thaumarchaeota could be involved in aerobic ammonia oxidation. OCHF Thaumarchaeota likely use the nitrite reductase (encoded by *nirK*) of the nitrogen monoxide-dependent ammonia oxidation pathway [48], since they lack the hydroxylamine dehydrogenase-encoding gene (*hao*).

## Conclusion

Using an integrated approach, we provide the first description of the microbial ecology of the recently discovered Old City hydrothermal field. So far, this site represents the first ‘Lost City’-type hydrothermal field observed along mid-ocean ridges. Therefore, OCHF is of particular interest to improve our understanding of ecosystems supported by oceanic serpentinization.

Despite the lack of environmental data, our results provide indirect evidences for slow and diffuse discharge of alkaline and reducing hydrothermal fluids at OCHF (i.e., characteristic carbonate-brucite assemblages forming chimneys, reduced predicted proteomes, potential anaerobic metabolisms involving various types of hydrogenases and the predominance of putative acetogens and formate- and CO-utilizing bacteria, among others). Dominant taxa and metabolisms vary between chimneys, in conjunction with the local redox state (±pH) evaluated from predicted proteomes. CO- and formate-based metabolisms are widespread, although CO and formate utilization as carbon or electron sources and related taxa may strongly differ from chimney to chimney. Although we provide a first insight into the metabolic capabilities and putative environmental conditions at OCHF, our study was hampered by technical limitations inherent to the unexpected discovery of deep oceanic hydrothermal sites. However, there is no doubt that future explorations and studies will give a better constrain of the metabolic potential of microbial lineages. We therefore encourage further investigations of this unique site, which could strongly improve our understanding of microbial ecology related to serpentinization. OCHF discovery also suggests that active ‘Lost City’-type hydrothermal fields could be more widespread on the seafloor than previously documented along mid-ocean ridges.

## Supplementary information

Supplementary Information
