# Supplementary material for: Microbial ecology of the newly discovered serpentinite-hosted Old City hydrothermal field (southwest Indian ridge)
Source: ISME J. 2020 Nov 2;15(3):818–32. doi: 10.1038/s41396-020-00816-7 (PMC8027613; doi:10.1038/s41396-020-00816-7)
Supplement: Supplementary file 1 — Supplementary Information [file 41396_2020_816_MOESM1_ESM.docx]

*Supplementary Information*

**Microbial ecology of the newly discovered serpentinite-hosted Old City hydrothermal field (southwest Indian ridge)**

Aurélien Lecoeuvre^1^, Bénédicte Ménez^1^, Mathilde Cannat^1^, Valérie Chavagnac^2^ and

Emmanuelle Gérard^1^

^1^ Université de Paris, Institut de physique du globe de Paris, CNRS UMR 7154, Paris, France

^2^ Université de Toulouse, Géosciences Environnement Toulouse, CNRS UMR 5563, Toulouse, France

**TABLE OF CONTENT**

**Supplementary Methods**

- Hydrothermal chimney sampling and archiving procedures
- Fluid sampling and archiving procedures
- Fluid analyses
- Chimney mineralogical and elemental characterization
- Confocal laser scanning microscopy
- Scanning electron microscopy
- DNA extractions
- Library preparation for 16S rRNA encoding genes and shotgun metagenomic sequencing
- Quality filtration of paired-end raw reads
- Near full length 16S rRNA encoding gene assembly and associated phylogenetic analysis
- Search for evidence of microbial H_2_ utilization and production

**Supplementary References**

**Supplementary Tables**

**Table S1.** Locations and primary characteristics of the chimneys sampled in this study.

**Table S2.** Potential contaminant genera removed from analyses.

**Table S3.** Metabolisms and associated key genes.

**Table S4.** Chemical characteristics of sampled fluids and ambient seawater.

**Table S5.** Major and trace element concentrations of chimney samples.

**Table S6.** Quality filtration results obtained on paired-end reads.

**Table S7.** Correlations between microbial communities’ diversity and mineralogical and chemical characteristics of chimney samples.

**Supplementary Figures**

**Figure S1-** Confocal laser scanning microscopy images of autoclaved *BaC* sample stained with Syto^TM^ 9.

**Figure S2-** Scanning electron microscopy images of chimney samples.

**Figure S3-** Relative abundance of the main ASV proteobacterial orders.

**Figure S4-** Relative abundance of microbial lineages in the metagenomes.

**Figure S5-** Maximum likelihood phylogenetic trees of Alpha- and Gammaproteobacteria based on near full-length 16S rRNA encoding gene reconstructed from metagenomic reads.

**Figure S6-** Maximum likelihood phylogenetic trees of Epsilonbacteraeota and Deltaproteobacteria based on near full-length 16S rRNA encoding gene reconstructed from metagenomic reads.

**Figure S7-** Maximum likelihood phylogenetic trees of Archaea based on near full-length 16S rRNA encoding gene reconstructed from metagenomic reads.

**Supplementary Methods**

*Hydrothermal chimney sampling and archiving procedures*

Five distinct hydrothermal chimneys (Figure 1) were sampled using the remotely operated vehicle (ROV) *Victor 6000* (Ifremer, France) in 15 l hermetic boxes previously filled with autoclaved distilled water filtered at 0.22 µm. The samples were then divided onboard into 4 subsamples in a laminar-flow hood using flame-sterilized pliers, scalpel and spatula. For the first subsample, several grams were collected in 50 ml Falcon^®^ tubes and immediately stored at -80°C prior to DNA extraction. For confocal laser scanning microscopy (CLSM), two subsamples were chemically fixed onboard with respectively 2% (vol/vol) formaldehyde in sterile seawater and with 50% (vol/vol) ethanol in sterile seawater following the protocol described elsewhere [1]. The formaldehyde fixation was conducted at 4°C for 3 h to allow impregnation of the bulk rock. The subsamples were then rinsed three times with sterile seawater and finally resuspended in 50% (vol/vol) ethanol in sterile seawater before storage at -20°C together with the ethanol-fixed subsamples. The last subsample was stored at 4°C without any treatment for scanning electron microscopy (SEM) observations or elemental and mineralogical characterizations.

*Fluid sampling and archiving procedures*

Fluid sampling was carried out by the PEP pumping fluid sampler mounted on the rear of the ROV *Victor 6000.* It enables fluid to be collected via a snorkel at temperatures below 120°C. However, at the time of sample collection, it was not possible to measure *in situ* the fluid temperature due to dysfunction of the ROV temperature probe. The PEP snorkel was positioned at putative chimney fluid outlets (i.e. generally on top of chimneys). However, no focused hydrothermal discharges in the form of plumes or shimmering fluids have been observed during operations, which made hydrothermal fluid sampling highly difficult. Between each fluid sampling, to avoid any contamination from previous sampling, the entire pumping system was purged with the surrounding seawater for at least 1 min, which corresponds to a time period longer than the pumping time for sample collection. Ambient seawater samples were also collected. Once the ROV was back on board, each 200 ml PEP bottle was disconnected from the carousel frame, brought to the laboratory and connected to a peristaltic pump for fluid extraction and filtration with a Sterivex^TM^ 0.22 µm filter. Fluid samples were then transferred to a low density polyethylene plastic bottle for storage in a cold room at 4°C. Fluid pH was measured immediately after extraction from the PEP bottles.

*Fluid analyses*

Elemental concentrations were measured by inductively coupled plasma-optical emission spectrometry (ICP-OES) using a Horiba Jobin Yvon Ultima 2 (Horiba Ltd., Japan) at the Géosciences Environnement Toulouse laboratory (Toulouse, France). The instrument was calibrated using IAPSO standard seawater (OSIL Limited, UK) [2] diluted up to 200 and achieves a precision of 2% or better. The analytical and instrumental procedures used for the analyses are described in details elsewhere [3, 4]. The full set of standards was run before and after each series of sample analyses to check the performance of the instrument. In addition, to assess possible instrument drift during analyses, one standard was run as a sample before, during and after each series of sample analyses.

*Chimney mineralogical and elemental characterization*

Semi-quantitative mineralogical analyses were carried out in triplicate on 300 mg of chimney powders by X-ray diffraction (XRD) at the Plateforme Rayons X (ITODYS, Université de Paris, France). Diffractograms (phi-scan with step of 0.1° 2θ for 10 h) were produced with a monochromatic Cu source using a Panalytical Empyrean diffractometer (Malvern Panalytical, Malvern, UK) equipped with a PIXcel multi-channel detector. Diffractograms were analyzed with the Panalytical XRD software by comparing the positions and intensities of the diffraction peaks against the software library of known crystalline materials. The chimney relative contents in minerals deduced from XRD analyses are provided with an uncertainty of ± 1wt%.

Major and trace element concentrations were analyzed in duplicate on 50 mg of chimney sample powders using ICP-MS at the Institut de physique du globe de Paris (IPGP, Université de Paris, France). For these measurements, the chimney samples were dissolved using a 1:1 mixture of 69% HNO_3_ and 50% HF acids in polypropylene tubes (SCP Science, Baie-d'Urfé, Canada) in a *Digi*PREP block digestor for elemental analysis. Concentrations were assessed using an Agilent 7900 quadrupole (Santa Clara, CA, USA) in pulse counting mode. For this purpose, dissolved samples were sprayed through a micro-nebulizer in a Scott spray chamber prior to ionization. Elements with atomic masses between that of sodium (23) and arsenic (75) were measured using a collision-reaction cell with helium gas (5 ml/min) to remove polyatomic interferences. All other elements were measured without collision gas.

*Confocal laser scanning microscopy*

Chemically-fixed chimney subsamples (as described in section *Hydrothermal chimney sampling and archiving procedures*) were stained with 10 µM of Syto^TM^ 9 green fluorescent nucleic acid dye (Thermo Fisher Scientific, Waltham, MA, USA) for 10 min in ice and washed with 1.5 ml of phosphate buffered saline 1x. Controls without staining were systematically performed on each chimney sample. In addition, in order to image stained chimney sample without intact DNA as an additional control, a fraction of *BaC* sample was autoclaved during 45 min at 121°C and then stained as described above.

CLSM images were obtained with concomitant excitation at 405 and 488 nm and fluorescence detection in the ranges of 425-475 and 500-530 nm using an Olympus FluoView FV1000 microscope (Tokyo, Japan) located at IPGP (Paris, France). CLSM image stacks were acquired, visualized and processed using the F10-ASW FluoView software (Olympus).

No fluorescence was observed in the Syto^TM^ 9 fluorescence emission range for unstained chimney samples and cell clusters were no longer visible in autoclaved *BaC* sample stained with Syto^TM^ 9 (Supplementary Figure S1).

*Scanning electron microscopy*

Chimney subsamples stored at 4°C or fixed with formaldehyde (as described in section *Hydrothermal chimney sampling and archiving procedures*) were cut into small fragments and air dried before SEM observations. In some instances, small quantities of sample were slightly grinded to exhume mineral encrusted biofilms. Samples were Au-coated before observations. SEM was carried out using a Zeiss AURIGA 40 field-emission scanning electron microscope (Oberkochen, Germany) located at IPGP. Images were acquired using secondary electron detectors (SESI detector for high and low current and InLens for low current) or with a backscattered electron detector (NTS BSD) with accelerating voltage ranging from 5 to 15 kV. Elemental analysis was carried out using an energy dispersive X-ray spectrometer Quantax 200 (125 eV resolution) from Bruker (Billerica, MA, USA).

*DNA extractions*

Samples were crushed in an ethanol-flame sterilized mortar and DNA was sequentially extracted from up to 0.5 g of sample powder each time. Due to the relative low biomass often associated with such environments, each bulk chimney sample was processed in several replicates pooled at the end of the purification step, although such approach precludes for any statistical analysis on sample replicates. Up to 2.5 g per chimney were used in total. Procedural blanks were systematically applied under the same conditions without samples. DNA concentrations were measured with the Qubit dsDNA high sensitivity kit (ThermoFisher Scientific) following the manufacturer’s recommendations.

*Library preparation for 16S rRNA encoding genes and shotgun metagenomic sequencing*

Tag sequencing of the 16S rRNA encoding gene was carried out in duplicate on an Illumina MiSeq platform at the Josephine Bay Paul Center for Comparative Molecular Biology and Evolution (Marine Biological Laboratory - MBL, Woods Hole Oceanographic Institution, MA, USA). The archaeal 517F/958R and bacterial 518F/926R fusion primers were used to target the V4-V5 hypervariable region with the protocols proposed by Nelson et al. (2014) [5]. For shotgun metagenomic sequencing, libraries were prepared with an insert size of about 400 bp following the manufacturer’s protocol of the NuGEN Ovation^®^ Ultralow V2 DNA-Seq kit (Tecan Genomics, Inc., Redwood City, CA, USA). Sequencing was then performed on an Illumina Nextseq500 instrument also at the MBL Josephine Bay Paul Center, leading to overlapped paired-end reads of 2 × 150 bp and using dedicated read indexing.

*Quality filtration of paired-end raw reads*

All demultiplexed paired-end raw reads were quality filtered following Minoche et al. (2011)’s recommendations [6]. For the 16S rRNA encoding gene amplicons, barcodes and primers were trimmed using Cutadapt 1.18 [7] allowing no errors. Then, quality filtered paired-end reads were merged with filtering at 5 mismatches over 100 nucleotides on the overlap region and at Phred quality of 30 using the illumina-utils tools [8]. Paired-end read filtering results are presented in Supplementary Table S6.

*Near full length 16S rRNA encoding gene assembly from metagenomes and associated phylogenetic analysis*

To perform phylogenetic analysis, small fragments of the 16S rRNA encoding genes in metagenomic high quality paired-end reads were identified using the SortMeRNA software v2.1 [9] against the SILVA database release 132 [10] internally indexed for Bacteria and Archaea. Extracted 16S rRNA encoding gene reads were subsequently mapped using bowtie [11] and assembled for each sample with EMIRGE [12-13] against the Silva database release 132 [10]. To this aim, we applied maximum lengths of 260 and 246 nucleotides for the read and the size of insert sequences, respectively. A standard deviation of 88 nucleotides for the insert sequence sizes and a 33 Phred quality were applied as recommended elsewhere (https://www.protocols.io/view/Detecting-16S-rRNA-Gene-Fragments-from-a-Metagenom-d7u9nv). We manually filtered the assembled near full length 16S rRNA encoding genes to remove sequences smaller than 1 200 bp or including ambiguities. The proportion of each phylotype was evaluated using the number of reads mapping to each of their respective sequences and normalized to the total number of identified reads.

Near full length 16S rRNA encoding gene sequences of each sample were aligned online with the SILVA Incremental Aligner SINA (https://www.arb-silva.de/aligner/) [14]. Results were imported in the ARB software [15] and merged with the full SILVA database release 132 [10]. Maximum likelihood phylogenetic trees were constructed based on the most abundant reconstructed 16S rRNA encoding gene sequences from our metagenomes and by collecting closely related environmental clones and isolated strains. Bootstrap support of the trees was based on 1 000 replicates.

*Search for evidence of microbial H_2_ utilization and production*

Genes encoding hydrogenases have recently been reclassified into groups and subgroups according to their phylogenetic position reflecting different physiologies [16-17]. However, some of these subgroups are not represented in the KEGG [18] and COG [19] database. Therefore, to assess hydrogenase diversity and relative abundance in our metagenomic dataset, we searched for hydrogenase specific domains in protein sequences obtained from Prodigal [20]. It was done against database constructed with sequences retrieved from the Pfam database [21-22] for [FeFe]-hydrogenases (PF02906) and [NiFe]-hydrogenases (PF00374). The database was constructed with the *hmmbuild* function and the specific domain was investigated with the *hmmsearch* function with an e-value cutoff of 10^-10^, all implemented in HMMER version 3.1 [23]. Then, to better constrain their function and abundance in chimney samples, identified hydrogenase protein sequences were submitted to the HydDB web tool [24] for further classification into groups and subgroups. To further characterize the functions of identified [FeFe]-hydrogenases (only identified at the groups level compared to subgroups for [NiFe]-hydrogenases), we searched for their enzyme annotations and protein homologies using PROKKA [25] and KEGG orthology [18], respectively. Note that the third type of hydrogenases, the [Fe]-hydrogenases was not found in our datasets. This type is restricted to some methanogens and is far less represented across environments [17].

**Supplementary References**

1. Gérard E, Ménez B, Couradeau E, Moreira D, Benzerara K, Tavera R, et al. Specific carbonate–microbe interactions in the modern microbialites of Lake Alchichica (Mexico). ISME J. 2013; 7: 1997–2009.

2. Besson P, Degboe J, Berge B, Chavagnac V, Fabre S, Berger G. Calcium, Na, K and Mg concentrations in seawater by inductively coupled plasma-atomic emission spectrometry: applications to IAPSO seawater reference material, hydrothermal fluids and synthetic seawater solutions. Geostand Geoanal Res. 2014; 38: 355–362.

3. Chavagnac V, Monnin C, Ceuleneer G, Boulart C, Hoareau G. Characterization of hyperalkaline fluids produced by low-temperature serpentinization of mantle peridotites in the Oman and Ligurian ophiolites. Geochem Geophys Geosyst. 2013; 14: 2496–2522.

4. Chavagnac V, Saleban Ali H, Jeandel C, Leleu T, Destrigneville C, Castillo A, et al. Sulfate minerals control dissolved rare earth element flux and Nd isotope signature of buoyant hydrothermal plume (EMSO-Azores, 37°N Mid-Atlantic Ridge). Chem Geol. 2018; 499: 111–125.

5. Nelson MC, Morrison HG, Benjamino J, Grim SL, Graf J. Analysis, optimization and verification of Illumina-generated 16S rRNA gene amplicon surveys. PLoS ONE 2014; 9: e94249.

6. Minoche AE, Dohm JC, Himmelbauer H. Evaluation of genomic high-throughput sequencing data generated on Illumina HiSeq and genome analyzer systems. Genome Biol. 2011; 12: R112.

7. Martin M. Cutadapt removes adapter sequences from high-throughput sequencing reads. EMBnet journal. 2011; 17: 10–12.

8. Eren AM, Vineis JH, Morrison HG, Sogin ML. A filtering method to generate high quality short reads using Illumina paired-end technology. PLoS ONE. 2013; 8: e66643.

9. Kopylova E, Noé L, Touzet H. SortMeRNA: fast and accurate filtering of ribosomal RNAs in metatranscriptomic data. Bioinformatics. 2012; 28: 3211–3217.

10. Quast C, Pruesse E, Yilmaz P, Gerken J, Schweer T, Yarza P, et al. The SILVA ribosomal RNA gene database project: improved data processing and web-based tools. Nucleic Acids Res. 2013; 41: D590–D596.

11. Langmead B, Trapnell C, Pop M, Salzberg SL. Ultrafast and memory-efficient alignment of short DNA sequences to the human genome. Genome Biol. 2009; 10: R25.

12. Miller CS, Baker BJ, Thomas BC, Singer SW, Banfield JF. EMIRGE: reconstruction of full-length ribosomal genes from microbial community short read sequencing data. Genome Biol. 2011; 12: R44.

13. Miller CS, Handley KM, Wrighton KC, Frischkorn KR, Thomas BC, Banfield JF. Short-read assembly of full-length 16S amplicons reveals bacterial diversity in subsurface sediments. PLoS ONE. 2013; 8: e56018.

14. Pruesse E, Peplies J, Glöckner FO. SINA: Accurate high-throughput multiple sequence alignment of ribosomal RNA genes. Bioinformatics. 2012; 28: 1823–1829.

15. Ludwig W, Strunk O, Westram R, Richter L, Meier H, Yadhukumar, et al. ARB: a software environment for sequence data. Nucleic Acids Res. 2004; 32: 1363–1371.

16. Boyd ES, Schut GJ, Adams MWW, Peters JW. Hydrogen metabolism and the evolution of biological respiration: Two separate families of enzymes that oxidize hydrogen and also produce it arose through convergent evolution. Microbe Magazine. 2014; 9: 361–367.

17. Greening C, Biswas A, Carere CR, Jackson CJ, Taylor MC, Stott MB, et al. Genomic and metagenomic surveys of hydrogenase distribution indicate H_2_ is a widely utilised energy source for microbial growth and survival. ISME J. 2016; 10: 761–777.

18. Kanehisa M, Sato Y, Morishima K. BlastKOALA and GhostKOALA: KEGG tools for functional characterization of genome and metagenome sequences. J Mol Biol. 2016; 428: 726–731.

19. Galperin MY, Makarova K S, Wolf YI, Koonin EV. Expanded microbial genome coverage and improved protein family annotation in the COG database. Nucleic Acids Res. 2014; 43: D261–D269.

20. Hyatt D, Chen G-L, Locascio PF, Land ML, Larimer FW, Hauser LJ. Prodigal: prokaryotic gene recognition and translation initiation site identification. BMC Bioinform. 2010; 11: 119.

21. Finn RD, Tate J, Mistry J, Coggill PC, Sammut SJ, Hotz H-R, et al. The Pfam protein families database. Nucleic Acids Res. 2008; 36: D281-288.

22. El-Gebali S, Mistry J, Bateman A, Eddy SR, Luciani A, Potter SC, et al. The Pfam protein families database in 2019. Nucleic Acids Res. 2019; 47: D427–D432.

23. Eddy SR. Accelerated profile HMM searches. PLoS Comput Biol. 2011; 7: e1002195.

24. Søndergaard D, Pedersen CNS, Greening C. HydDB: A web tool for hydrogenase classification and analysis. Sci Rep. 2016; 6: 34212.

25. Seemann T. Prokka: rapid prokaryotic genome annotation. Bioinformatics. 2014; 30: 2068–2069.

26. Sheik CS, Reese BK, Twing KI, Sylvan JB, Grim SL, Schrenk MO, et al. Identification and removal of contaminant sequences from ribosomal gene databases: Lessons from the Census of Deep Life. Front Microbiol. 2018; 9: 840.

27. Millero FJ, Feistel R, Wright DG, McDougall TJ. The composition of Standard Seawater and the definition of the Reference-Composition Salinity Scale. Deep Sea Res Part I Oceanogr Res Pap. 2008; 55: 50–72.

28. Suzuki S, Ishii S, Hoshino T, Rietze A, Tenney A, Morrill PL, et al. Unusual metabolic diversity of hyperalkaliphilic microbial communities associated with subterranean serpentinization at The Cedars. ISME J. 2017; 11: 2584–2598.

29. Creevey CJ, Doerks T, Fitzpatrick DA, Raes J, Bork P. Universally distributed single-copy genes indicate a constant rate of horizontal transfer. PLoS ONE. 2011; 6: e22099.

30. Brazelton WJ, Schrenk MO, Kelley DS, Baross JA. Methane- and sulfur-metabolizing microbial communities dominate the Lost City hydrothermal field ecosystem. Appl Environ Microbiol. 2006; 72: 6257–6270.

31. Brazelton WJ, Ludwig KA, Sogin ML, Andreishcheva EN, Kelley DS, Shen C-C, et al. Archaea and bacteria with surprising microdiversity show shifts in dominance over 1,000-year time scales in hydrothermal chimneys. Proc Natl Acad Sci. USA. 2010; 107: 1612–1617.

**Supplementary Tables**

**Table S1.** Locations and primary characteristics of the chimneys sampled in this study. mbsl, meters below sea level; IGSN, International Geo Sample Number (http://www.geosamples.org/igsnabout).

| **Sample**  **name** | **Sample**  **abbreviation** | **Latitude** | **Longitude** | **Depth**  **(mbsl)** | **IGSN code** |
| --- | --- | --- | --- | --- | --- |
| RS-644-12 | *BaC* | -27,8428 | 64,5893 | 3185 | CNRS0000001139 |
| RS-644-28 | *PiMo* | -27,8430 | 64,5882 | 3223 | CNRS0000001155 |
| RS-649-20 | *Chan* | -27,8406 | 64,5911 | 3062 | CNRS0000001287 |
| RS-644-25 | *OCT* | -27,8436 | 64,5938 | 3136 | CNRS0000001152 |
| RS-649-24 | *ToMo* | -27,8414 | 64,5882 | 3120 | CNRS0000001291 |

**Table S2.** Potential contaminant genera [26] removed from analyses.

| **Phylum** | | **Class** | | | **Contaminant genera** | | |
| --- | --- | --- | --- | --- | --- | --- | --- |
| Proteobacteria | | Alphaproteobacteria | | *Asticcacaulis* | | |  |
|  |  |  |  | *Bradyrhizobium* | | |  |
|  |  |  |  | *Caulobacter* | | |  |
|  |  |  |  | *Devosia* | | |  |
|  |  |  |  | *Methylobacterium* | | |  |
|  |  |  |  | *Rhizobium* | | |  |
| Firmicutes |  | | | *Bacillus* | | |  |
|  |  |  |  | *Paenibacillus* | | |  |
| Actinobacteria |  | | | *Arthrobacter* | | |  |
|  |  |  |  | *Rhodococcus* | | |  |
| Bacteroidetes |  | | | *Dyadobacter* | | |  |
|  |  |  |  | *Flavobacterium* | | |  |
|  |  | | *Pedobacter* | | |  |  |

**Table S3.** Metabolisms and associated key genes with their accession number in the KEGG database [18] or Enzyme Commission number (EC). Note that the classification of hydrogenases was computed by HydDB [24]. Corresponding KEGG accession numbers are provided for information purposes.


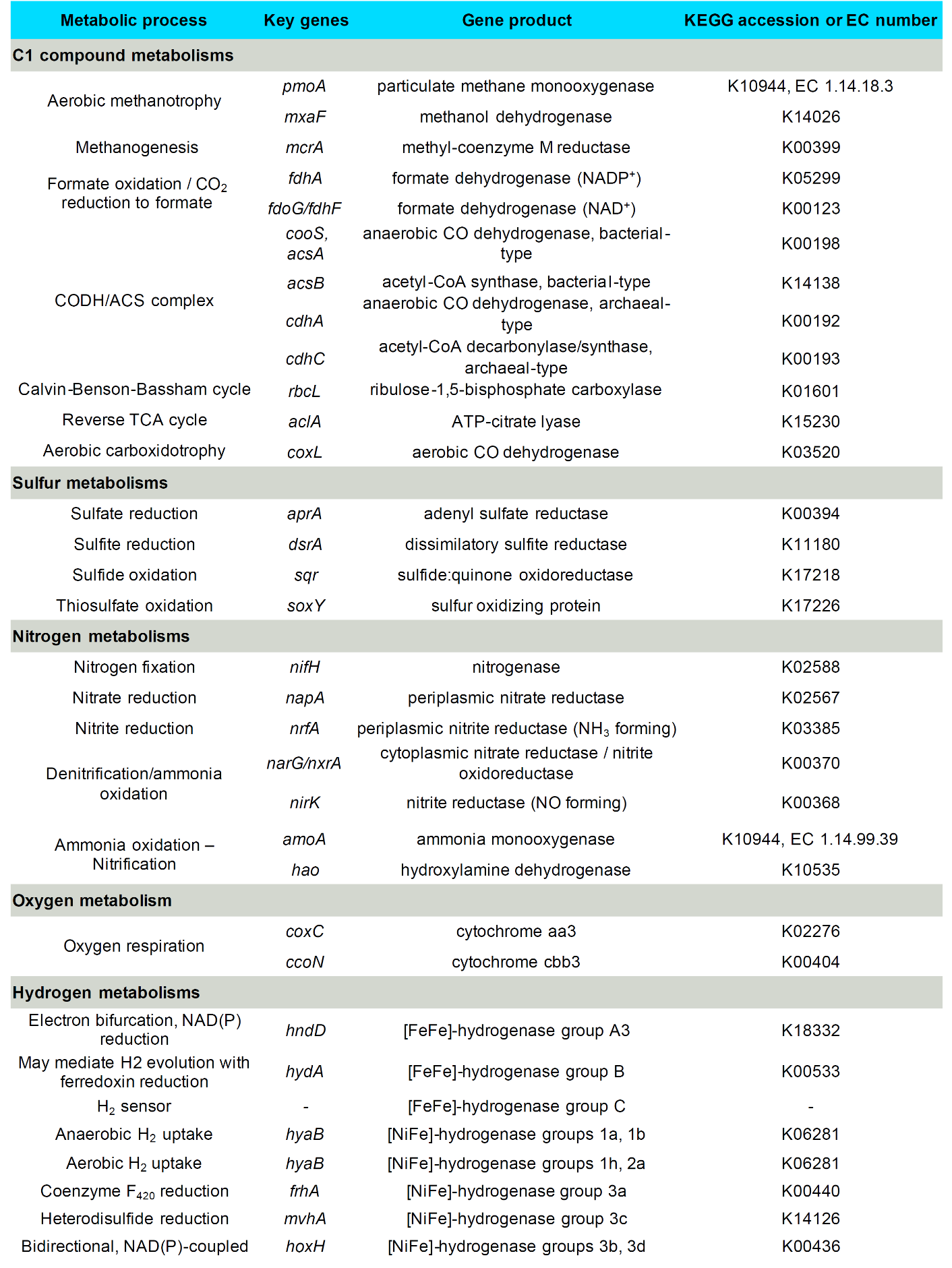


**Table S4.** Chemical characteristics of fluids sampled as close as possible to putative chimney fluid outlets and of ambient seawater. Element concentrations were measured by ICP-OES. Standard seawater values are from [27].

**Table S5.** Major and trace element concentrations of chimney samples measured by ICP-MS. The standard deviation calculated from chemical standards for each replicate is indicated in % under brackets. ppm, parts per million; c.d., close to detection limit; b.d., below detection.

**Table S6.** Quality filtration results obtained on paired-end reads (Supplementary Methods). Tag sequencing of the 16S rRNA encoding genes was performed in duplicate. n.d., not detected; ORFs, open read frames.

**Table S7.** Correlations based on Mantel tests between microbial communities’ diversity estimated by the Bray-Curtis distance and the mineralogical and chemical characteristics of chimney samples.

**Supplementary Figures**


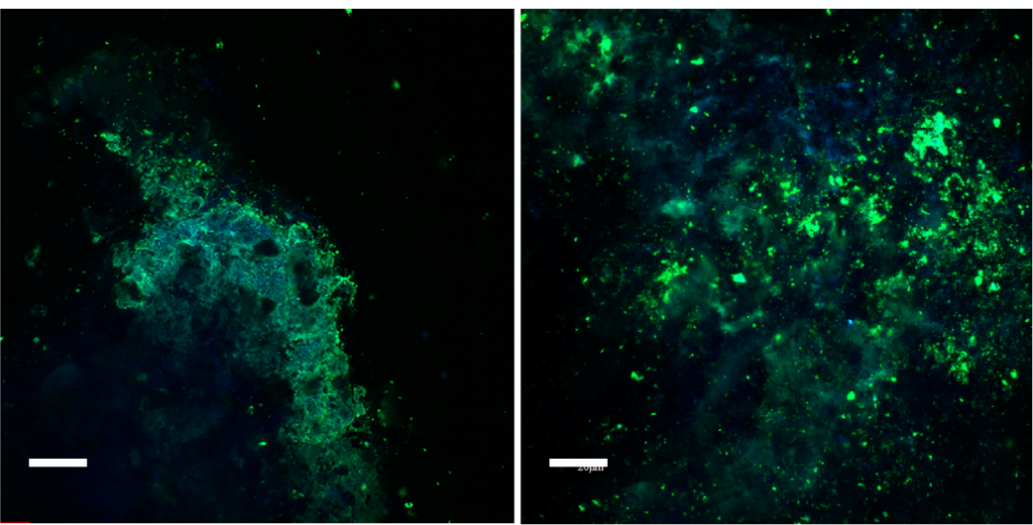


**Figure S1-** Confocal laser scanning microscopy images of autoclaved *BaC* sample stained with Syto^TM^ 9. The green fluorescent dye stains the surface of the minerals where DNA is adsorbed as a result of cell lysis during autoclaving. Scale bar 20 µm.

**Figure S2-** Scanning electron microscopy images of chimney samples highlighting clues for microbial colonization. Images show the different structures harbored by chimneys. (a) C-rich filaments (as indicated by energy dispersive X-ray spectrometry) likely representing extracellular polymeric substances (EPS) coating brucite; (b) coccoid-like structures; (c) filaments fully encrusted in brucite; (d) carbon-rich filaments coated by nanocrystalline brucite; (e) EPS among brucite crystals; (f) carbonate crystals. Images (a) and (c-f) were acquired in backscattered electron mode at 15 kV and image (b) in secondary electron mode at 5 kV.

**Figure S3-** Relative abundance of the main ASV proteobacterial orders in the hydrothermal chimney samples. cand., candidate phylum.

**Figure S4-** Relative abundance of microbial lineages in the metagenomes collected in the 5 hydrothermal chimneys studied. This has been evaluated based on NCBI taxonomic annotation of 12 or 40 single-copy genes (SCGs) shared between all lineages [28-29], on the reconstructed near full-length 16S rRNA encoding genes using Silva database released 132 [10], and on all genes, according to the lowest common ancestor (LCA) algorithm. Unclassified Bacteria and Archaea are not represented. Phyla that do not account for more than 1% of all genes in at least one chimney sample are merged as “Other”. Corresponding taxonomy in the Silva database release 132 (as used in Figures 2 and 4 and Supplementary Figures S5-S7): Bipolaricaulota = Acetothermia; cand. NPL-UPA2 = candidate phylum BHI80-139; Omnitrophica = Omnitrophicaeota; Epsilonproteobacteria = Epsilonbacteraeota.

*It should be noted that SCGs and reconstructed near full length 16S rRNA encoding genes taxonomic annotations are not based on the same database, which may explain the relative differences in lineage abundance profiles. Moreover, SCGs, like all metagenomic genes, are annotated based on LCA algorithm and, as a result, a relatively high proportion (~20%) of genes are classified as Bacteria, Archaea or Proteobacteria without deeper classification. Therefore, LCA-based approach may influence the relative abundance of microbial lineages, particularly for lineages that are poorly represented in databases. Aware of these biases, results nevertheless show high consistency with all the different approaches carried out on metagenomic genes, as well as with the microbial community compositions estimated from 16S rRNA encoding gene amplicons (Figure 2). Among the few differences, we note the higher relative abundance for all samples of Patescibacteria in both SCGs-based profiles. Chloroflexi, Actinobacteria and Bipolaricaulota (formerly Acetothermia) are relatively more abundant in BaC and PiMo based on metagenomic approaches compared to the amplicons one.*

**Figure S5-** Maximum likelihood phylogenetic trees of Alpha- and Gammaproteobacteria based on near full-length 16S rRNA encoding gene reconstructed from metagenomic reads. To highlight phylotype diversity, only abundtant sequences (> 1.5%) are represented. Sequences from this study are colored with respect to chimney samples (Supplementary Table S1) with normalized relative abundance in brackets (%). Environmental clone and cultivated isolate accession numbers are indicated in brackets with serpentinization-related ecosystems in bold. They correspond to the closest hits to the near full-length 16S rRNA encoding gene sequences reported in this study using the SILVA database release 132 [10]. Bootstrap values over 70% support based on 1 000 replicates are indicated by black dots on their respective nodes. LCHF, Lost City hydrothermal field; EPR, East Pacific rise; GoM, Gulf of Mexico; SWIR, southwest Indian ridge; MMG2 and MMG3, marine methylotrophic group 2 and 3.

**Figure S6-** Maximum likelihood phylogenetic trees of Epsilonbacteraeota and Deltaproteobacteria based on near full-length 16S rRNA encoding gene reconstructed from metagenomic reads. To highlight phylotype diversity, only abundant sequences (> 1%) are represented. Sequences from this study are colored with respect to chimney samples (Supplementary Table S1) with normalized relative abundance in brackets (%). Environmental clone and cultivated isolate accession numbers are indicated in brackets with serpentinization-related ecosystems in bold. They correspond to the closest hits to the near full-length 16S rRNA encoding gene sequences reported in this study using the SILVA database release 132 [10]. Bootstrap values over 70% support based on 1 000 replicates are indicated by black dots on their respective nodes. LCHF, Lost City hydrothermal field; EPR, East Pacific rise; ELSC; East Lau spreading center; JdFR, Juan de Fuca ridge.

**Figure S7-** Maximum likelihood phylogenetic tree of Archaea based on near full-length 16S rRNA encoding gene reconstructed from metagenomic reads. To highlight phylotype diversity, only abundant sequences (> 1%) are represented. Sequences from this study are colored with respect to chimney samples (Supplementary Table S1) with normalized relative abundance in brackets (%). Environmental clone and cultivated isolate accession numbers are indicated in brackets with serpentinization-related ecosystems in bold. They correspond to the closest hits to the near full-length 16S rRNA encoding gene sequences reported in this study using the SILVA database release 132 [10]. The LCMS sequences are collected from [30-31]. Bootstrap values over 70% support based on 1 000 replicates are indicated by black dots on their respective nodes. LCHF, Lost City hydrothermal field; LCMS, Lost City Methanosarcinales; TCMS, The Cedars Methanosarcinales.
